# Analytical validation of whole exome and whole genome sequencing for clinical applications

**DOI:** 10.1186/1755-8794-7-20

**Published:** 2014-04-23

**Authors:** Michael D Linderman, Tracy Brandt, Lisa Edelmann, Omar Jabado, Yumi Kasai, Ruth Kornreich, Milind Mahajan, Hardik Shah, Andrew Kasarskis, Eric E Schadt

**Affiliations:** 1 Icahn Institute for Genomics and Multiscale Biology, Icahn School of Medicine at Mount Sinai, New York, NY, USA; 2 Department of Genetics and Genomic Sciences, Icahn School of Medicine at Mount Sinai, New York, NY, USA

## Abstract

**Background:**

Whole exome and genome sequencing (WES/WGS) is now routinely offered as a clinical test by a growing number of laboratories. As part of the test design process each laboratory must determine the performance characteristics of the platform, test and informatics pipeline. This report documents one such characterization of WES/WGS.

**Methods:**

Whole exome and whole genome sequencing was performed on multiple technical replicates of five reference samples using the Illumina HiSeq 2000/2500. The sequencing data was processed with a GATK-based genome analysis pipeline to evaluate: intra-run, inter-run, inter-mode, inter-machine and inter-library consistency, concordance with orthogonal technologies (microarray, Sanger) and sensitivity and accuracy relative to known variant sets.

**Results:**

Concordance to high-density microarrays consistently exceeds 97% (and typically exceeds 99%) and concordance between sequencing replicates also exceeds 97%, with no observable differences between different flow cells, runs, machines or modes. Sensitivity relative to high-density microarray variants exceeds 95%. In a detailed study of a 129 kb region, sensitivity was lower with some validated single-base insertions and deletions “not called”. Different variants are "not called" in each replicate: of all variants identified in WES data from the NA12878 reference sample 74% of indels and 89% of SNVs were called in all seven replicates, in NA12878 WGS 52% of indels and 88% of SNVs were called in all six replicates. Key sources of non-uniformity are variance in depth of coverage, artifactual variants resulting from repetitive regions and larger structural variants.

## Background

Whole exome and genome sequencing (WES/WGS) is now routinely offered as a clinical test by a growing number of laboratories. WES/WGS is implemented as a laboratory-developed test that must be fully validated by the offering laboratory prior to use. This validation effort “establishes the analytical performance for the clinical test system … to confirm that the system is suitable for its intended use” [1], and in this context (next-generation sequencing) is focused on three inter-related aspects: platform, test-specific and informatics pipeline validation.

This report documents one such validation of WES/WGS for patients without a molecular diagnosis but suspected constitutional disease mutation(s), with attention to parameters that measure the reproducibility of the testing platform as well as the informatics pipeline. We performed a focused evaluation of the analytical performance characteristics of SNV and small indel (less than 50 bp) detection for a single workflow across multiple technical replicates. This study complements the comparisons of different sequencing technologies [2,3], exome capture techniques [4,5] and informatics pipelines [6,7] that have been reported previously.

The American College of Medical Genetics (ACMG) has developed clinical laboratory standards for NGS [8], which specifically address the unique challenges of WES/WGS. Since WES/WGS is not targeting specific diseases/genes, the validation effort is not focused on the specific sequence contexts and variant types associated with those diseases, but rather on developing and evaluating end-to-end metrics for high quality sequencing. Different expectations apply to WES/WGS than targeted panels or single gene testing. For instance, instead of “sequence-to-completion” for a specific region the goal is to sensitively and precisely call variants over the largest percentage of the target region possible, while also being able to determine and report which bases did not meet the minimum requirements for successful variant calling.

The variant calling error rate is determined by many parameters; setting a single minimum threshold for confident calls is challenging. We do not attempt to define a fixed set of quality filters that produce our desired sensitivity and specificity. Instead we use variant quality score recalibration (VQSR) [9,10], a statistical technique for variant filtration that builds a model of “true” variants using multiple quality parameters and then applies that model to filter out likely false positive variants. With VQSR we set desired end-to-end sensitivity and determine the thresholds for specific quality parameters directly from the data itself. All performance characteristics were measured in the context of this filtering approach. VQSR can be combined with separate filters used to flag for additional investigation genomic regions with increased likelihood of missed/artifactual variants or genotypes with increased likelihood of incorrect zygosity.

The scale of WES/WGS makes it prohibitive to evaluate all variants with alternative technologies and the cost limits the number of samples that can be sequenced as part of the validation process. We sequenced multiple technical replicates of five reference samples in a scheme to extensively but efficiently test intra-run, inter-run, inter-machine and inter-mode reproducibility. We compared this data to a variety of reference callsets including SNP arrays, Sanger validation data from targeted NGS panels offered by our laboratory and publicly available variant calls to evaluate accuracy, sensitivity and specificity. Ongoing standardization efforts are continually producing additional and improved reference materials (RMs) and associated callsets [11]; in this report, for example, we use the first release of the “Genomes In a Bottle” (GIAB) variant callset [12]. The rapid evolution of sequencing technology, informatics tools and RMs make validation and optimization a continuous process in which these results represent a particular moment in time. The process we describe, however, is largely automated and readily adaptable to new informatics tools and data resources as they become available.

## Methods

### Materials

**Figure 1:**
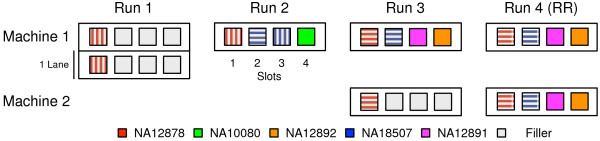
**Schematic of sequencing runs used in WES validation experiments.** Up to 4 samples are multiplexed into a single lane high-throughput lane. Cross-hatching indicates the same or different library preparations. Run #4 is an Illumina HiSeq 2500 RapidRun, with each lane treated as a separate replicate. Individual replicates are named as *sample/run-machine-slot.*

**Figure 2:**
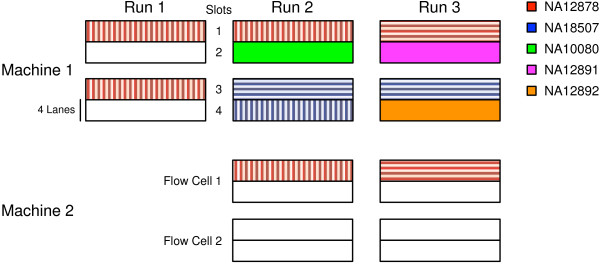
**Schematic of sequencing runs used in WGS validation experiments.** Cross-hatching indicates the same or different library preparations. Individual replicates are named as *sample/run-machine-slot.*

The samples listed in Table 1 were sequenced as multiple technical replicates according to the schematics shown in Figures 1 and 2. The samples were chosen to include a trio (NA12878-NA12891-NA12892) with numerous reference callsets both public and internal, ethnic diversity (NA18507) and a sample (NA10080) with a known disease mutation and internal reference materials. The replicates were designed to test intra-run (same run of same machine), inter-run (different run of same machine), inter-library, inter-machine and inter-mode (between high-throughput and rapid run modes) reproducibility. Note that each pair-wise comparison between replicates may represent more than one of the above comparisons; tables of comparison type are included in the Additional file 1. Replicates are named by *sample/run-machine-slot.* DNA was derived from lymphoblastoid cell lines from the Coriell Institute for Medical Research.

**Table 1:** Summary of samples used in validation experiments

**Sample**	**Sex**	**Known variants**	**Note**
NA12878	F	Heterozygous *CYP2C19* c.681G > A (rs4244285)	
NA12891	M	Homozygous *CYP2C19* c.681G > A (rs4244285)	Father to NA12878
NA12892	F	N/A	Mother to NA12878
NA10080	M	Heterozygous *PTEN* c.781C > T	
NA18507	M	N/A	

Known variants are those reported by Coriell.

Table 2 lists the reference materials used in this analysis.

**Table 2:** Description of reference callsets used in analysis

**Dataset**	**Notes (version, target intervals, etc.)**
In-house Omni 2.5 Microarray	Described in methods section
1000G Omni 2.5 Microarray	As distributed in GATK Resource Bundle version 2.3
Hapmap	Version 3.3. As distributed in GATK Resource Bundle version 2.3
Genome in a Bottle	Version 2.17, dated Oct. 17 2013. Most restrictive “high confidence” intervals excluding simple repeats, segmental duplications, decoys, STRs, and known CNVs.
Autism(ASD) Panel	129 kilobase targeted clinical sequencing panel of genes related to austism spectrum disorder (ASD). Indels are left aligned.

### Sequencing and variant calling

WES and WGS was performed on a HiSeq 2000/2500 (Illumina, San Diego, CA, USA) with a 100 base-pair (bp) paired-end protocol. WES samples were barcoded and pooled with up to three other samples prior to enrichment for exonic DNA with the Nimblegen SeqCap EZ Human Exome Library v3.0. The sequencing data is available via the SRA under BioProjects PRJNA241071 and PRJNA241062.

The genome analysis pipeline (GAP) is based on the 1000 Genomes Project (1000G) data analysis data pipeline and is composed from the widely used open source software projects bwa 0.7.5a [13], Picard 1.96 [14], GATK 2.7 [9,10], snpEff 3.0 [15], BEDTools 2.16.2 [16] and custom-developed software. Short-reads are aligned to a gender- and pseudo-autosomal region (PAR)-masked build of the hg19 human reference genome using bwa mem. The GAP implements the “GATK Best Practices” including indel realignment, de-duplication, and base-quality score recalibration (BQSR).

**Figure 3:**
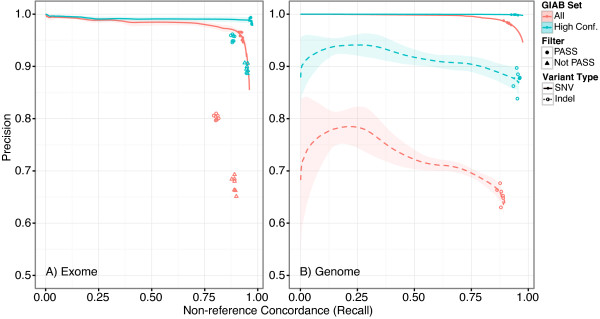
**Precision vs. non-reference concordance (NRC) for WES (A) and WGS (B) SNVs (solid line) and Indels (dashed line) as a function of VQSR VQSLoD score relative to GIAB high confidence and all variant sets.** Thick line is mean across all replicates; shaded region shows the standard deviation. Points show the PR at the VQSR PASSing threshold. VQSR is not applied in WES; points show PR for PASSing and all variants (both PASS and not-PASS).

Single nucleotide variants (SNVs) and indels were called jointly with the GATK HaplotypeCaller. Variant quality score recalibration (VQSR) was used to estimate the probability that a WES or WGS SNV is a true variant instead of an artifact and set the corresponding variant filter thresholds. VQSR was used for WGS but not WES indels, due to an insufficient number of variants to train the model; fixed filters were used instead. The PASS threshold for VQSR is set to capture of 99.5% known true-positives. We observed this threshold to offer a good compromise between precision and recall; the impact of changing this threshold can be observed in Figure 3 in the context of the Genome in a Bottle (GIAB) reference material. Note that in choosing a threshold below 100% we set a corresponding minimum false negative rate.

The exome capture targets were expanded with 100 bp flanks for variant calling. Mean coverage and factions of bases at different coverage levels were calculated with the un-flanked intervals; the callable coverage of RefSeq coding exons was calculated with the flanked intervals.

### Array genotyping

Array genotyping was performed on 750 nanograms of DNA with the Human Omni2.5 BeadChip (Illumina, San Diego, CA, USA). The hybridized arrays were scanned using the HiScan system (Illumina, San Diego, CA, USA) and the genotypes called utilizing the software GenomeStudio v1.0 Genotyping Module (Illumina, San Diego, CA, USA).

### Validation statistics

**Figure 4:**
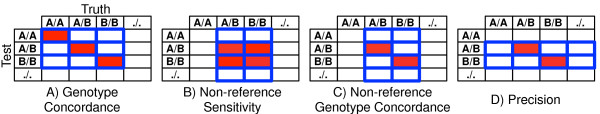
**Different genotype concordance metrics.** ‘B’ is the alternate allele. Each metric is calculated as the ratio of red elements to blue-outline elements. Non-reference genotype concordance (NRC) is genotype-aware sensitivity/recall.

Genotype concordance (concordance) and the related metrics, non-reference sensitivity (NRS), non-reference genotype concordance (NRC) and precision are computed as the ratio of solid red elements to blue outline elements shown in Figure 4 (where “B” is the non-reference allele). Non-PASSing variants are treated as “no-calls” (./.) and multi-allelic sites are decomposed into their component bi-allelic variants. Callsets are joined using strict variant equality (position, reference and alternate alleles); thus different representations of the same complex variant will not be recognized as concordant. However, wherever relevant (ASD panel callset) all variants have been similarly left-aligned to reduce this effect. And we note that the GIAB callset was regularized with the same variant caller (GATK Haplotype Caller). Where relevant, and unless otherwise specified, all metrics are computed with respect to the target intervals associated with the callset e.g. the target intervals for any gene panel and the “high-confidence” intervals supplied alongside the GIAB callset.

All metrics are sensitive to the choice of variants in the truth and test sets, especially the concordance metric, which is determined by the intersection of the “test” and “truth” datasets. Including many homozygous reference genotypes, which can be easier to call, can bias the concordance metric. The GAP only reports variants from the reference, minimizing the biasing effect of homozygous reference calls. Similarly, restricting the variants in the test set to only very high quality variants will also bias the concordance statistic, as would restricting the variants included in the truth set to those readily called in NGS data. All NGS replicates use the filtering scheme described previously, and any filters applied to the reference materials are specified.

Although the SNP microarrays and other callsets are treated as “truth”, they are not error free. The error rate for the BeadArray technology is estimated at 0.3% [17], although the actual error rate will be a function of the QC strategy employed and as the data shown here suggests, is actually higher, than that estimate in this context. There is no “truth” callset when comparing two WES or WGS callsets that should be identical. In these cases we conduct the same concordance analysis twice, treating each callset alternately as both “truth” and “test”. By definition the concordance is symmetric, but differences in NRS, NRC and Precision will be observed.

### Experiments

Table 3 summarizes the different validation experiments performed. Metrics are computed as described previously. The WES and WGS replicates used in different experiments are listed in Table 4, with all replicates used for concordance testing against alternate technologies and curated variant sets.

**Table 3:** Summary of validation experiments performed

**Experiment**	**Action**
**Concordance with SNP Array**	Measure concordance with high-density SNP arrays
**Concordance with integrated NGS-derived variants**	Measure concordance with Genome In A Bottle (GIAB) NA12878 callset
**Calling variants from targeted NGS panel**	Measure concordance with calls from a targeted NGS panel (all calls previously validated either by in-house Sanger assays or the presence of the variant in the sample in Hapmap, 1000G, etc.)
**Intra-run reproducibility**	Measure concordance for the same sample sequenced in the same run with:
	A) Different sample preparations across the same flow cell, or
	B) The same sample preparation across different flow cells
**Inter-run reproducibility**	Measure concordance for the same sample on the same machine with:
	A) The same sample preparation different runs, or
	B) Different sample preparation across different runs
**Inter-machine reproducibility**	Measure concordance for the same sample with the same sample preparation on different machines of the same model in the same run cycle
**Inter-mode reproducibility**	Measure concordance between high-throughput and rapid run Illumina modes

**Table 4:** WES and WGS samples used in different concordance experiments

**Experiment**	**WES comparison sets**	**WGS comparison sets**
**Intra-run reproducibility**	NA12878: r1-1-1 vs. r1-1-2	NA12878: r1-1-1 vs. r1-1-3
	NA18507: r2-1-2 vs. r2-1-3	NA18507: r2-1-3 vs. r2-1-4
**Inter-run reproducibility**	NA18507: r2-1-2 vs. r3-1-2	NA18507: r2-1-3 vs. r3-1-3
	NA18507: r2-1-3 vs. r3-1-2	NA18507: r2-1-4 vs. r3-1-3
	NA12878: r1-1-1 vs. r2-1-1 vs. r3-1-1	NA12878: r1-1-1 vs. r2-1-1 vs. r3-1-1
		NA12878: r2-2-1 vs. r3-2-1
**Inter-machine reproducibility**	NA12878: r3-1-1 vs. r3-2-1	NA12878: r3-1-1 vs. r3-2-1
	NA12878: r4-1-1 vs. r4-2-1	NA12878: r2-1-1 vs. r2-2-1
	NA18507: r4-1-2 vs. r4-2-2	
**Inter-mode reproducibility**	NA12878, NA18507, NA12891, NA12892 r4-*-* versus all others	N/A

## Results and discussion

### Sequencing statistic

Tables 5 and 6 list the coverage statistics for the different WES and WGS replicates, respectively. The percentage of RefSeq coding bases considered confidently callable represents an estimate of the portion of those bases over which we could confidently call variants. Confidence is determined by: a minimum of 20-fold coverage and no more than 10% MAPQ0 (ambiguously mapped) reads. This estimate is intended to be conservative (the coverage threshold is set at the upper end of the range suggested in the guidelines [8]) however, we may still fail to call variants in regions that are considered “confidently callable”.

**Table 5:** Summary of WES coverage statistics

**Replicate**	**Mean**	**% Bases > =1X**	**% Bases > =10X**	**% Bases > =20X**	**% Bases > =30X**	**% Coding bases callable**
NA12878						
r1-1-1	57.4	96.8	93.6	89.2	79.3	83.8
r1-1-2	57.4	96.8	93.6	89.1	79.3	83.8
r2-1-1	66.8	96.2	92.0	85.4	75.2	79.7
r3-1-1	81.8	96.7	93.6	90.7	85.1	84.8
r3-2-1	74.5	96.7	93.6	90.8	85.0	85.1
r4-1-1	83.7	96.7	93.7	90.9	85.5	85.0
r4-2-1	73.6	96.6	93.2	89.1	81.5	83.1
NA12891						
r3-1-3	70.9	96.5	92.4	86.0	75.8	80.1
r4-1-3	70.8	96.6	92.5	85.9	75.7	80.2
r4-2-3	60.9	96.3	91.4	82.2	69.8	76.5
NA12892						
r3-1-4	70.7	96.5	92.7	87.5	78.7	82.5
r4-1-4	71.4	96.6	92.8	87.7	79	82.7
r4-2-4	61.7	96.4	91.9	84.5	73.2	79.7
NA18507						
r2-1-2	66.4	96.4	92.1	84.7	73.8	78.7
r2-1-3	92.2	96.7	93.6	90.9	86.2	84.9
r3-1-2	81.2	96.7	93.2	88.9	81.3	82.9
r4-1-2	82.8	96.7	93.3	89.2	81.7	83.2
r4-2-2	71.5	96.5	92.6	86.4	76.6	80.4
NA10080						
r2-1-4	60.7	96.4	92.1	84.5	73.2	78.4

Percent RefSeq Coding Bases Callable reports the percentage of all RefSeq coding exons bases (as downloaded from the UCSC Genome Browser) considered callable. The baseline value for the capture targets is 95.5%.

**Table 6:** Summary of WGS coverage statistics

**Replicate**	**Mean**	**% Bases > =1X**	**% Bases > =10X**	**% Bases > =20X**	**% Bases > =30X**	**% Coding bases callable**
NA12878						
r1-1-1	51.0	95.4	94.9	94.3	93.1	91.8
r1-1-3	49.8	95.4	94.9	94.3	93.1	91.9
r2-1-1	36.5	95.4	94.7	93.5	86.9	91.6
r2-2-1	34.6	95.4	94.7	93.2	82.9	91.2
r3-1-1	49.9	95.5	95.0	94.5	93.5	91.9
r3-2-1	39.8	95.5	94.8	93.9	89.4	91.0
NA12891						
r3-1-2	42.7	96.8	96.1	94.3	88.7	91.2
NA12892						
r3-1-4	45.1	95.2	90.8	86.6	81.3	90.9
NA18507						
r2-1-3	30.0	96.8	95.7	89.4	65.8	87.3
r2-1-4	35.8	96.8	96.0	92.3	83.4	90.2
r3-1-3	51.7	96.9	96.3	95.4	91.6	91.6
NA10080						
r2-1-2	35.1	96.9	96.1	92.0	81.7	89

Percent RefSeq Coding Bases Callable reports the percentage of all RefSeq coding exons bases (as downloaded from the UCSC Genome Browser) considered callable.

### Microarrays

**Figure 5:**
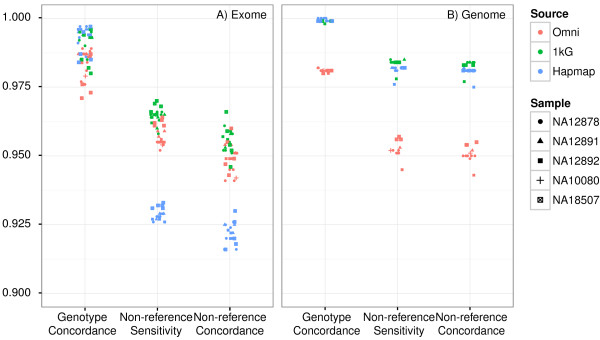
**WES and WGS genotype concordance (concordance), non-reference sensitivity (NRS) and non-reference concordance (NRC) relative to three different SNP microarray genotypes: 1) an Illumina Omni2.5 genotype performed in-house, 2) an Omni2.5 genotype performed as part of the 1000 Genomes project, and 3) Hapmap 3.3 genotype.** Not all genotypes are available for all samples. Only those SNPs within the exome capture targets are considered for WES concordance. The GAP does not report non-variant sites, so homozygous reference calls are not considered in the concordance evaluation.

Figure 5 shows the concordance, NRS and NRC relative to three different SNP microarray genotypes (the “In-house Omni 2.5 Microarray”, “1000G Omni 2.5 Microarray” and “Hapmap” reference callsets). The concordance exceeds 97% (98.5% for more filtered 1000G array), with the NRS exceeding 92.5% (95% excluding HapMap). These results are consistent with the concordance rates listed in the guidelines [8]. The observed NRS is lower than the sensitivity set in VQSR (99.5%), even though the same datasets (1000G Omni 2.5 and Hapmap 3.3 genotypes) are used in that training process, showing the impact of insufficient coverage and various error processes, e.g. low complexity and structural variants, on end-to-end sensitivity. For example, more missed variants are located on hyper-variable MHC region of chromosome 6, than any other chromosome region. The NRS increases 2-3% if we restrict the concordance analysis to those variants in regions identified as callable per the requirements specified above.

The stratification in NRS and NRC reflects the different technologies and QC procedures of the array data sources. More filtering was applied to the 1000G Omni 2.5 genotypes by its providers [18], such as flagging SNVs within 20 bp of an indel, than the Omni 2.5 genotyping performed in-house, which has no specific QC filters applied. We expect higher NRS and NRC relative to the 1000G arrays as a result. Unlike the Omni arrays, the HapMap dataset was lifted to hg19 from hg18/GRCh36. The resulting mapping errors could introduce artifactual variants that bias sensitivity.

### Genome in a bottle

The mean concordance, NRS and NRC relative to the GIAB variant callset (with full range in brackets), restricted to variants within the GIAB high-confidence regions, are 99.0% [98.2,99.6], 96.1% [95.4,96.5] and 95.1% [94.5,95.8], respectively, for WES and 99.7% [99.6,99.7], 94.2% [93.0,95.4] and 93.9% [92.7,95.1], respectively, for WGS. All metrics are improved compared to using the microarrays as the truth callset, as would be expected. The GIAB callset is produced from similar NGS datasets, using a similar analysis pipeline, and the analysis was restricted to the GIAB high-confidence regions. If we further restrict to those variants in regions identified in each replicate as callable (and thus eliminate low coverage regions in each sample), the mean NRS increases to 98.9% and 95.0% for WES and WGS respectively, with the NRS improving for all replicates. Approximately 90% of those WGS variants that would be considered FNs relative to the GIAB callset are called in our data, but marked as non-PASSing. MQ is identified as the “culprit” by VQSR in the majority of those filtered variants. We note that MQ is no longer a recommended VQSR annotation, a change we would expect to improve VQSR performance.

To investigate the impact of our filtering approach on analytical performance, we plot the precision-recall (PR) curves for WES/WGS SNVs and indels relative to the all and just the high-confidence GIAB callsets in Figure 3. The points show actual PR at the PASS threshold. We observe that the filters are effectively set at the inflection point where relaxing the threshold results more false positives (FPs) than true positives (TPs). We note that for SNVs in the GIAB high-confidence region the rate is approximately 1:1, i.e. roughly equal numbers of FPs and TPs result from relaxed filtering. In situations, such as clinical workflows, where FNs are very problematic and FPs less so, treating all variants in the high-confidence region as PASSing might be a good tradeoff.

### Targeted gene panel

xTable 7 lists the site-level sensitivity (NRS), specificity and genotype-level NRC relative to Sanger validated and or HapMap, 1000G and NIST genotypes in 129 kilobases (kb) of target from a clinical multigene panel (the ASD Panel callset). Not all variants were validated during panel design and testing, e.g. intronic variants or the Sanger results are inconclusive so we cannot always determine if a WES variant is a false positive (FP). Thus we report a mix of FPs and “excess” positives, variants discovered with NGS that were not Sanger confirmed or not conclusively reported elsewhere.

**Table 7:** Site-level sensitivity (NRS), specificity and genotype-level NRC relative to Sanger validated and or Hapmap or 1000G-reported variants in 129 Kb of target from a clinical gene panel (the ASD panel callset)

**Replicate**	**Variants**	**TP**	**FP/EP**	**FN**	**TN**	**Diff. Alleles**	**Sensitivity (NRS)**	**NRC**
NA12878 Exome								
r1-1-1	48	40	5	3	0	0	93.0%	81.4%
r1-1-2	46	40	2	3	1	0	93.0%	83.7%
r2-1-1	45	37	1	6	1	0	86.0%	74.4%
r3-1-1	44	40	0	3	1	0	93.0%	83.7%
r3-2-1	44	40	0	3	1	0	93.0%	83.7%
r4-1-1	44	39	0	4	1	0	90.7%	79.1%
r4-2-1	44	39	0	4	1	0	90.7%	81.4%
NA12878 Genome								
r1-1-1	64	54	1	8	1	1	87.3%	81.0%
r1-1-3	74	53	11	9	1	1	85.7%	79.4%
r2-1-1	73	53	10	9	1	1	85.7%	77.8%
r2-2-1	73	54	10	8	1	1	87.3%	81.0%
r3-1-1	71	55	8	7	1	1	88.9%	81.0%
r3-2-1	64	52	1	10	1	1	84.1%	74.6%

Not all variants in this interval were validated, e.g. intronic variants, so we cannot conclusively determine if a variant is a false positive. Instead we report “excess positives”, variants discovered in the NGS replicates that were not Sanger confirmed or conclusively reported elsewhere.

Table 8 lists the false negative (FN) variants for NA12878 in the panel’s target regions. The most common FN, 3/6 WES FNs and 9/11 WGS FNs, is a single-base deletion in a homopolymer region, e.g. a poly-A tract. The sequencing instrument’s error rate increases in homopolymer regions, making it more difficult to detect indels in these contexts [19,20]. As such, these FNs are not unexpected. For these particular variants, the deletion is typically detected in a small fraction of the reads (<10%), too few to accurately call the heterozygous deletion. A pileup for a representative example is included in the Additional file 1. Increasing the read depth does reduce the number FNs that result from variation in filtering, but not the missed deletions. Most of the single-base deletions are also not reliably called in high-depth (over 250-fold) targeted sequencing. As in the WES/WGS, the deletions are detected in only a small fraction of the reads.

**Table 8:** False negative variants in NA12878 relative to the ASD panel callset

**Position**	**Variant**	**Truth Genotype**	**Called In**	**Would be Reviewed**	**Note**
**Exome**					
chr12:2614070	G > T	G/T	6/7	Yes (silent)	Called, but filtered in one replicate
chrX:15863648	GA > G	GA/G	0/7	No	Not called in any replicate; 10 bp homopolymer
chrX:135115669	GA > G	GA/G	0/7	No	Called, but filtered in two replicates; 11 bp homopolymer
chrX:152954025	A > G	G/G	6/7	Yes (UTR)	Low depth region
chrX:153287314	TG > T	TG/T	0/7	Yes (UTR)	Called, but filtered in all replicates for QD; 10 bp homopolymer
Genome					
chr5:176639217	TA > T	TA/T	0/6	No	Not called in any replicate; 13 bp homopolymer
chr7:146805220	AT > A	AT/A	0/6	No	Not called in any replicate; 11 bp homopolymer
chr10:89720633	CT > C	CT/C	0/6	No	Not called in any replicate; 15 bp homopolymer
chr10:89720907	T > G	T/G	0/6	No	Called, but filtered in all replicates
chr11:70348852	G > CG	CG/CG	0/6	No	Called, but as heterozygous in all replicates; inside 12 bp homopolymer
chrX:15863648	GA > G	GA/G	0/6	No	Not called in any replicate; 10 bp homopolymer
chrX:132888207	TA > T	TA/T	3/6	No	Not called in three replicates; 16 bp homopolymer
chrX:135067675	G > C	G/C	5/6	Yes (missense)	Not called in one replicate
chrX:135115669	GA > G	GA/G	1/6	No	Called in one replicate; 11 bp homopolymer
chrX:153287314	TG > T	TG/T	1/6	Yes (UTR)	Called in one replicate; 10 bp homopolymer
chrX:153357614	TA > T	TA/T	1/6	No	Called in one replicate; 13 bp homopolymer

Most common error mode is single base deletions in homopolymer regions. Each variant is annotated as to whether it would be further reviewed in interpretations workflow (Yes) or automatically filtered out from further consideration (No).

The FN variants in Table 8 impact the analytical sensitivity, but may not necessarily impact clinical sensitivity. The variants chrX:15863648GA > G and chrX:135115669GA > G, for example, occur in homopolymer intronic regions outside the invariant splice site, and thus are unlikely to result in a change in protein functionality and would not be considered for in-depth review. We annotated each variant in Table 8 as to whether it would be reviewed in a variant interpretation workflow focused exclusively on known disease mutations or exonic/splice variants; 50% or more variants would be filtered out automatically and not reviewed further.

To evaluate our sensitivity, more generally, for the subset of variants more likely to be pathogenic, we tested the sensitivity relative to the high-confidence loss-of-function (LoF) mutations in NA12878 reported in MacArthur et al.’s study of LoF variants in the 1000 Genomes cohort [21]. We detected 56–57 of 61 variants in the WES replicates (sensitivity of 92-93%) and 68–70 of 73 variants in the WGS replicates (sensitivity of 93-96%). As with other NGS-derived callsets, directly extrapolating sensitivity from this dataset is likely an overestimate (because the dataset may not include variant types/regions that are difficult to sequence).

In those NA12878 replicates with multiple FPs relative to the ASD panel callset, the majority are artifacts created by two larger structural variants in the introns of *SHANK3* detected manually during the review of discordant calls. Although the individual variants are PASSing, manual review of the pileup (Additional file 1) immediately shows these variants to be artifacts of the larger variant. The same variant caller can produce very different calls for different sequencing replicates, even with similar coverage, in and around these structural variants and repeat regions. The differences are not in the filtering, but in which variants the caller emits. These artifactual variants manifest as FPs, and will reduce the NRS and NRC, but not the concordance, when using NGS datasets as truth. The remaining FPs are non-recurrent errors in 1–2 replicates.

### Technical replicates

**Figure 6:**
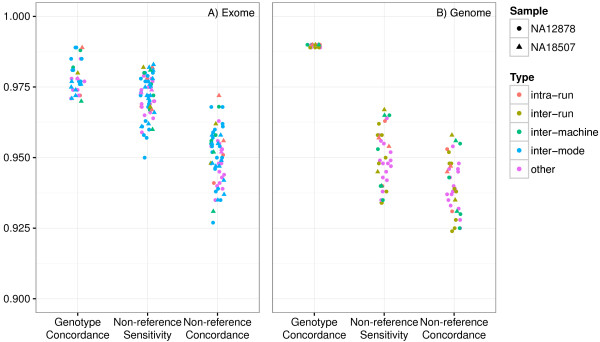
**WES and WGS genotype concordance (concordance), non-reference sensitivity (NRS) and non-reference concordance (NRC) for all pairwise comparisons of the technical replicates, differentiated by the kind of comparison: intra-run, inter-run, inter-machine and inter-mode.** Those comparisons marked as “other” fit into multiple categories, including inter-library. Each replicate is alternately treated as both “truth” and “test”.

Figure 6 shows the concordance, NRS and NRC for all pairwise comparisons of NA12878 and NA18507 replicates, marked as the kind of comparison, i.e. intra-run, inter-run, inter-machine, inter-mode, inter-library, where appropriate. Many comparisons are of more than one kind; those without a clear primary specific classification are marked as “other”. Visual inspection does not indicate any clear relationship between comparison kind and concordance. To quantitatively assess the contribution of these different kinds of comparisons to the concordance, we performed a multiple linear regression analysis of the five different comparison kinds as binary variables and the sample ID as a binary control variable on concordance for all NA12878 and NA18507 replicate pairs. A separate regression analysis was performed for WES (31 pairs) and WGS (18 pairs). WES did not significantly differ from the null model (sample covariate alone) at a threshold of 0.05, while WGS did significantly differ (P = 0.016). We further analyzed each WGS comparison type individually. The only comparison kind to be reported as significant (P = .0044) at a threshold of 0.0125 (Bonferroni corrected for 4 tests) was WGS inter-library; the coefficient estimate translates to a reduction in concordance of 0.0006, i.e. from 99% to 98.94%. The regression analysis is described in more detail in the Additional file 1.

Table 9 lists the fractions of different types of variants uniformly called in different fractions of replicates. Of all variants identified in WES data from the NA12878 reference sample 74% of indels and 89% of SNVs were called PASSing or not PASSing in all seven replicates. In NA12878 WGS 51% of indels and 88% of SNVs were uniformly called in all six replicates. The non-uniformity is only partially a result of variation in depth of coverage. Restricting the analysis to the “squared-off” intersection of confidently callable regions across all replicates increases the percentage of SNVs in all replicates by approximately 2–4 and indels by 7–10. Further restricting the analysis to the GIAB high-confidence regions further increases the percentages of variants uniformly called across all replicates by a similar amount: 89.5% of indels and 97.9% of SNVs are uniformly called in the NA12782 WES replicates, and 71.0% of indels and 93.2% of SNVs in the WGS replicates. The number of uniquely observed variants, i.e. variants called in only one replicate, is in the range [118–252] and [29395–54746] for NA12878 WES and WGS, respectively, and [182–335] and [66342–104224] for NA18507 WES and WGS respectively.

**Table 9:** Percentage of variants, by type, that are called identically at the site level across different subsets of replicates

							
**NA12878 WES**	**1/7**	**2/7**	**3/7**	**4/7**	**5/7**	**6/7**	**7/7**
SNV	3.20	1.37	1.18	1.28	1.42	2.69	88.85
Indel	10.25	4.60	2.62	2.33	2.51	3.71	73.99
NA18507 WES	1/5	2/5	3/5	4/5	5/5		
SNV	2.87	1.60	1.47	2.62	91.43		
Indel	7.61	3.57	2.96	3.70	82.17		
NA12878 WGS	1/6	2/6	3/6	4/6	5/6	6/6	
SNV	3.40	1.76	1.52	1.79	3.69	87.80	
Indel	20.20	9.89	6.11	5.17	7.07	51.60	
NA18507 WGS	1/3	2/3	3/3				
SNV	2.61	2.93	94.46				
Indel	20.01	13.10	66.89				

N/N indicates the variant site, but not necessarily genotype, was identified in all replicates.

## Conclusions

We evaluated the intra-run, inter-run, inter-mode and inter-machine reproducibility, concordance with orthogonal technologies (microarray, Sanger) and sensitivity relative to known variant sets (GIAB, MacArthur et al.) for both WES and WGS across multiple technical replicates of five different reference samples. These analyses were performed as part of the validation of WES/WGS as a clinical test in our laboratory and as a result focus on a single informatics pipeline and exome capture technology. However since both technologies are widely used or are generally reflective of alternative approaches, the results presented here should be broadly relevant to anyone interested in the performance characteristics of WES/WGS.

The state of reference callsets is changing rapidly, with GIAB and GeT-RM being just two examples of newly available callsets for NA12878. Those and similar resources will continue to improve and expand, and therefore any analysis using such resources represents a snapshot of a particular moment in time. The different sensitivities observed for different kinds of callsets (microarray, NGS-derived datasets like GIAB and Sanger sequencing) shows the continued challenges in genotyping different kinds of variation as well as the continuing challenges in building high-quality, comprehensive reference callsets The comparisons with Sanger data, for instance, show that indels in homopolymer regions are under-detected, while the comparisons to NGS-derived datasets, like GIAB, show how the structural variants and repeat regions can be interpreted very differently by different variant callers, even when restricting to high-coverage, high-confidence regions.

At the time of this report, there are no absolute standards for the analytical performance characteristics for WES/WGS but the results presented here fall within the ACMG suggested ranges [8]. By the nature of WES/WGS, most of the reported analyses are focused on analytical performance characteristics that are independent of any particular clinical scenario. We have successfully used the documented workflow, however, to make a molecular diagnosis in 4 of 8 pilot clinical WES cases, indicating that its performance translates to a useful clinical sensitivity that is on par with that reported by other laboratories [22] and at the suggested 50% threshold for cost effectiveness [23]. The difference between the clinical sensitivity and the much higher analytical sensitivity for SNVs and small indels reported here reinforces the need to continue to improve our sequencing, variant calling and variant interpretation technologies (even a 1% false negative rate in WES/WGS translates into many variants in absolute terms) and our ability to identify other variant types, e.g. CNVs, which contribute to disease burden.

## Abbreviations

SNV: Single nucleotide variant; WES: Whole exome sequencing; WGS: Whole genome sequencing; GAP: Genome analysis pipeline; RM: Reference material; Concordance: Genotype concordance; NRS: Non-reference sensitivity; NRC: Non-reference concordance; GIAB: Genome in a bottle.

## Competing interests

The Mount Sinai Genetic Testing Laboratory offers fee-for-service whole exome sequencing. The authors declare to no other competing interests.

## Authors’ contributions

MDL implemented the genome pipeline, designed and performed the analyses and drafted the manuscript. TB designed the analysis and performed the Sanger validation of the targeted regions. HS performed the primary analysis of the sequencing data. LE, RK and TB designed the number and types of sequencing runs and supervised the clinical aspects of the validation. MM, OJ, and YK supervised and performed the sequencing. EES and AK supervised the sequencing and pipeline development/deployment. All authors read and approved the final manuscript.

## Pre-publication history

The pre-publication history for this paper can be accessed here:

http://www.biomedcentral.com/1755-8794/7/20/prepub

## Supplementary Material

Additional file 1: Analytical Validation of Whole Exome and Whole Genome Sequencing for Clinical Applications. Click here for file

## References

[B1] Gargis AS Kalman L Berry MW Bick DP Dimmock DP Hambuch T Lu F Lyon E Voelkerding KV Zehnbauer BA Agarwala R Bennett SF Chen B Chin ELH Compton JG Das S Farkas DH Ferber MJ Funke BH Furtado MR Ganova-Raeva LM Geigenmüller U Gunselman SJ Hegde MR Johnson PLF Kasarskis A Kulkarni S Lenk T Liu CSJ Manion M Assuring the quality of next-generation sequencing in clinical laboratory practice Nat Biotechnol 2012 7 1033 1036 10.1038/nbt.2403 23138292 PMC3827024

[B2] Rieber N Zapatka M Lasitschka B Jones D Northcott P Hutter B Jäger N Kool M Taylor M Lichter P Pfister S Wolf S Brors B Eils R Coverage bias and sensitivity of variant calling for four whole-genome sequencing technologies PLoS One 2013 7 e66621 10.1371/journal.pone.0066621 23776689 PMC3679043

[B3] Lam HYK Clark MJ Chen R Chen R Natsoulis G O’Huallachain M Dewey FE Habegger L Ashley EA Gerstein MB Butte AJ Ji HP Snyder M Performance comparison of whole-genome sequencing platforms Nat Biotechnol 2012 7 78 82 10.1038/nbt.2065 PMC4076012 22178993

[B4] Asan Xu Y Jiang H Tyler-Smith C Xue Y Jiang T Wang J Wu M Liu X Tian G Wang J Wang J Yang H Zhang X Comprehensive comparison of three commercial human whole-exome capture platforms Genome Biol 2011 7 R95 10.1186/gb-2011-12-9-r95 21955857 PMC3308058

[B5] Clark MJ Chen R Lam HYK Karczewski KJ Chen R Euskirchen G Butte AJ Snyder M Performance comparison of exome DNA sequencing technologies Nat Biotechnol 2011 7 908 914 10.1038/nbt.1975 21947028 PMC4127531

[B6] O’Rawe J Jiang T Sun G Wu Y Wang W Hu J Bodily P Tian L Hakonarson H Johnson WE Wei Z Wang K Lyon GJ Low concordance of multiple variant-calling pipelines: practical implications for exome and genome sequencing Genome Med 2013 7 28 10.1186/gm432 23537139 PMC3706896

[B7] Liu X Han S Wang Z Gelernter J Yang B-Z Variant callers for next-generation sequencing data: a comparison study PLoS One 2013 7 e75619 10.1371/journal.pone.0075619 24086590 PMC3785481

[B8] Rehm HL Bale SJ Bayrak-Toydemir P Berg JS Brown KK Deignan JL Friez MJ Funke BH Hegde MR Lyon E ACMG clinical laboratory standards for next-generation sequencing Genet Med 2013 7 733 747 10.1038/gim.2013.92 23887774 PMC4098820

[B9] DePristo MA Banks E Poplin R Garimella KV Maguire JR Hartl C Philippakis AA del Angel G Rivas MA Hanna M McKenna A Fennell TJ Kernytsky AM Sivachenko AY Cibulskis K Gabriel SB Altshuler D Daly MJ A framework for variation discovery and genotyping using next-generation DNA sequencing data Nat Genet 2011 7 491 498 10.1038/ng.806 21478889 PMC3083463

[B10] McKenna A Hanna M Banks E Sivachenko A Cibulskis K Kernytsky A Garimella K Altshuler D Gabriel S Daly M DePristo MA The genome analysis toolkit: a MapReduce framework for analyzing next-generation DNA sequencing data Genome Res 2010 7 1297 1303 10.1101/gr.107524.110 20644199 PMC2928508

[B11] Zook JM Salit M Genomes in a bottle: creating standard reference materials for genomic variation - why, what and how? Genome Biol 2011 7 Suppl 1 31 10.1186/gb-2011-12-s1-p31

[B12] Zook JM Chapman B Wang J Mittelman D Hofmann O Hide W Salit M Integrating human sequence data sets provides a resource of benchmark SNP and indel genotype calls Nat Biotechnol 2014 advance on 10.1038/nbt.2835 24531798

[B13] Li H Durbin R Fast and accurate short read alignment with Burrows-Wheeler transform Bioinformatics 2009 7 1754 1760 10.1093/bioinformatics/btp324 19451168 PMC2705234

[B14] Picard Team Picard 2012

[B15] Cingolani P Platts A Wang LL Coon M Nguyen T Wang L Land SJ Lu X Ruden DM A program for annotating and predicting the effects of single nucleotide polymorphisms, SnpEff: SNPs in the genome of Drosophila melanogaster strain w1118; iso-2; iso-3 Fly (Austin) 2012 7 80 92 10.4161/fly.19695 22728672 PMC3679285

[B16] Quinlan AR Hall IM BEDTools: a flexible suite of utilities for comparing genomic features Bioinformatics 2010 7 841 842 10.1093/bioinformatics/btq033 20110278 PMC2832824

[B17] Shen R Fan J-B Campbell D Chang W Chen J Doucet D Yeakley J Bibikova M Wickham Garcia E McBride C Steemers F Garcia F Kermani BG Gunderson K Oliphant A High-throughput SNP genotyping on universal bead arrays Mutat Res 2005 7 70 82 10.1016/j.mrfmmm.2004.07.022 15829238

[B18] Abecasis GR Auton A Brooks LD DePristo MA Durbin RM Handsaker RE Kang HM Marth GT McVean GA An integrated map of genetic variation from 1,092 human genomes Nature 2012 7 56 65 10.1038/nature11632 23128226 PMC3498066

[B19] Albers CA Lunter G MacArthur DG McVean G Ouwehand WH Durbin R Dindel: accurate indel calls from short-read data Genome Res 2011 7 961 973 10.1101/gr.112326.110 20980555 PMC3106329

[B20] Highnam G Franck C Martin A Stephens C Puthige A Mittelman D Accurate human microsatellite genotypes from high-throughput resequencing data using informed error profiles Nucleic Acids Res 2013 7 e32 10.1093/nar/gks981 23090981 PMC3592458

[B21] MacArthur DG Balasubramanian S Frankish A Huang N Morris J Walter K Jostins L Habegger L Pickrell JK Montgomery SB Albers CA Zhang ZD Conrad DF Lunter G Zheng H Ayub Q DePristo MA Banks E Hu M Handsaker RE Rosenfeld JA Fromer M Jin M Mu XJ Khurana E Ye K Kay M Saunders GI Suner M-M Hunt T A systematic survey of loss-of-function variants in human protein-coding genes Science 2012 7 823 828 10.1126/science.1215040 22344438 PMC3299548

[B22] Yang Y Muzny DM Reid JG Bainbridge MN Willis A Ward PA Braxton A Beuten J Xia F Niu Z Hardison M Person R Bekheirnia MR Leduc MS Kirby A Pham P Scull J Wang M Ding Y Plon SE Lupski JR Beaudet AL Gibbs RA Eng CM Clinical whole-exome sequencing for the diagnosis of Mendelian disorders N Engl J Med 2013 131002140031007 10.1056/NEJMoa1306555 PMC4211433 24088041

[B23] Shashi V McConkie-Rosell A Rosell B Schoch K Vellore K McDonald M Jiang Y-H Xie P Need A Goldstein DG The utility of the traditional medical genetics diagnostic evaluation in the context of next-generation sequencing for undiagnosed genetic disorders Genet Med 2013 10.1038/gim.2013.99 23928913

