# Supplementary material for: Analytical validation of whole exome and whole genome sequencing for clinical applications
Source: BMC Med Genomics. 2014 Apr 23;7:20. doi: 10.1186/1755-8794-7-20 (PMC4022392; doi:10.1186/1755-8794-7-20)
Supplement: Additional file 1: — Analytical Validation of Whole Exome and Whole Genome Sequencing for Clinical Applications. [file 1755-8794-7-20-S1.pdf]

Analytical Validation of Whole Exome and Whole Genome Sequencing for Clinical Applications

Michael D. Linderman[1,2], Tracy Brandt[2], Lisa Edelmann[2], Omar Jabado[1,2], Yumi Kasai[1,2], Ruth Kornreich[2], Milind Mahajan[1,2], Hardik Shah[1,2], Andrew Kasarskis[1,2], Eric E Schadt[1,2]

[1]Icahn Institute for Genomics and Multiscale Biology, [2]Dept. of Genetics and Genomic Sciences Icahn School of Medicine at Mount Sinai, New York, NY

Supplementary Methods and Results

Sequencing Protocol

The DNA is sheared by focused energy to a 250bp average size fragment for WES and a 300bp average size fragment for WGS. Adaptor-ligated DNA fragments are amplified by PCR and purified to generate the gDNA sequencing library.

WES: The gDNA library is barcoded and pooled with up to three other exome samples from the same or similar projects, i.e. up to four-way multiplexed. The pooled samples are enriched for exonic DNA with the SeqCap EZ Human Exome Library v3.0 (Nimblegen), which offers 64Mb of sequence capture that covers greater than 95% of genes in RefSeq. The pooled, enriched gDNA library is amplified by PCR. The resulting gDNA library is clustered on the HiSeq Flow Cell (Illumina) and sequenced with a 100bp paired-end protocol to obtain approximately 40-50 million paired-end reads per sample.

WGS: The gDNA library is clustered on four of the eight lanes of the HiSeq "High Output" FlowCell (Illumina) and sequenced with a 100bp paired-end protocol to obtain approximately 600-800 million paired-end reads per sample.

Alignment, Variant Calling, Variant Annotation, Sequencing Metrics

The genome analysis pipeline (GAP), shown in Figure 1, is based on the 1000 Genomes data analysis pipeline [1, 2] and is composed from the widely-used open source software projects bwa 0.7.5a[3], Picard 1.96[4], GATK 2.7[1, 2], snpEff 3.0[5], BEDTools 2.16.2[6], PLINK/SEQ and custom-developed software.

FASTQ files containing all reads that pass instrument quality control are generated from the BCL files produced by the Illumina HiSeq instrument using Casava 1.8.2. If multiple samples are multiplexed into a single instrument lane, these samples are de-multiplexed during FASTQ generation.

Short reads are aligned using bwa mem to a gender- and pseudo-autosomal regions (PAR)-masked build of the hg19 human reference genome as distributed in version 2.3 of the GATK resource bundle. The PAR are masked on chromosome Y, and all of chromosome Y is masked for samples known to be 46XX. After the initial alignment local multiple sequence realignment is performed for reads spanning known or suspected indels, molecular duplicates are removed and base quality score recalibration (BQSR) is applied (ReadGroup, QualityScore, Cycle and Context covariates).

Single nucleotide variants (SNVs) and indels are called jointly with the GATK HaplotypeCaller. For WES and WGS variant quality score recalibration is used to estimate the probability that a SNV is a true polymorphism instead of an artifact and set the corresponding variant filter thresholds. For

WGS, where more data is available, VQSR is also used for insertion and deletions; for WES fixed filters are applied since fewer variants are available (as recommended in the GATK Best Practices).

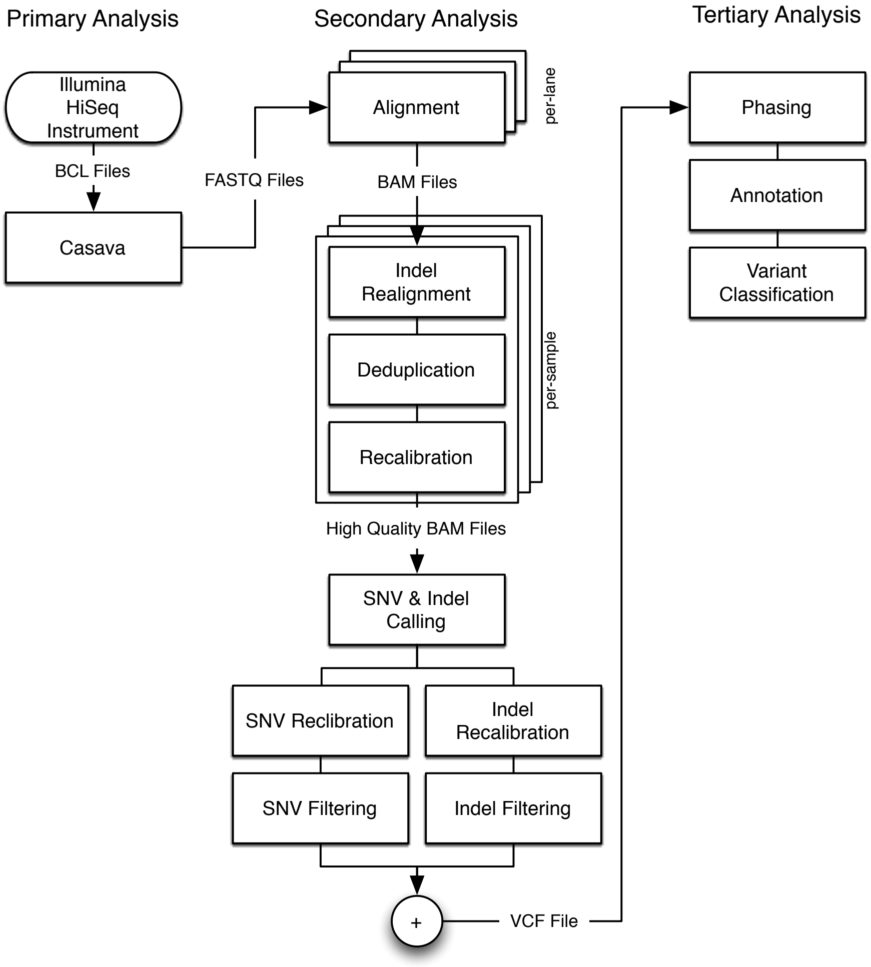

Figure 1: Schematic of genome analysis pipeline

The specific metrics used in VQSR are summarized in Table 1. Following the GATK best practices, dbSNP, HapMap3.3, Mills/1000 Genomes indels and 1000 Genomes Illumina Omni2.5 SNP microarray datasets (all distributed as part of the GATK resource bundle) are used as the training data for VQSR.

Table 1: Summary of VQSR annotations in use in the GAP

Annotation	SNV	Indel	Note
**QD**	×	×	Quality by depth
**MQ**	×		Mapping quality
**MQRankSum**	×		Mann-Whitney Rank Sum Test (MWW) for mapping qualities for reads with reference vs. alternate allele
**ReadPosRankSum**	×	×	MWW for distance to the end of the read for reads with references vs. alternate allele
**FS**	×	×	Fisher's Exact Test of variant strand bias
**DP**	×	×	Total unfiltered depth over all samples. Note: Not used with hybrid selection samples due to highly variable coverage

	depth.

The products of VQSR are a VQSLOD score (the log odds ratio of being a true variant) for each variant and a set of VQSLOD score thresholds, or tranches, that aim to capture a specific proportion of true positives. Each tranche is less specific but more sensitive, introducing additional true positive calls along with additional false positive calls. We set the PASSing threshold at 99.5%, i.e. we set VQSLOD threshold to the value estimated by VQSR to capture that percentage of true positives. We have observed this threshold to offer a good compromise between precision and recall. In choosing a threshold below 100%, however, we set a corresponding minimum false negative rate.

Genotype concordance (concordance), non-reference sensitivity (NRS), non-reference concordance (NRC) and precision are computed as described in the main text using a modified version of the GATK GenotypeConcordance walker. Site-level sensitivity and specificity is calculated using a modified version of the GATK VariantEval ValidationReport module.

Comparison to Sanger Results in 129kb Targeted ASD Panel

Tables 7 and 8 in the main text describe the false-positive and false-negative variants observed relative to the ASD Panel callset. The pileup for representative examples are included below.

Figure 2 shows the pileup across NA12878 WGS replicates for a representative false negative variant. This variant chr5:176639217TA>T is one of several heterozygous single base-pair deletions in homopolymer regions not called in the NGS data. The deletion is clearly observable, but in small (< 10%) fraction of reads at the site.

Figure 3 shows the pileup across WGS replicates for one of two structural variants in SHANK3. This structural variant is a source of false positive variant calls.

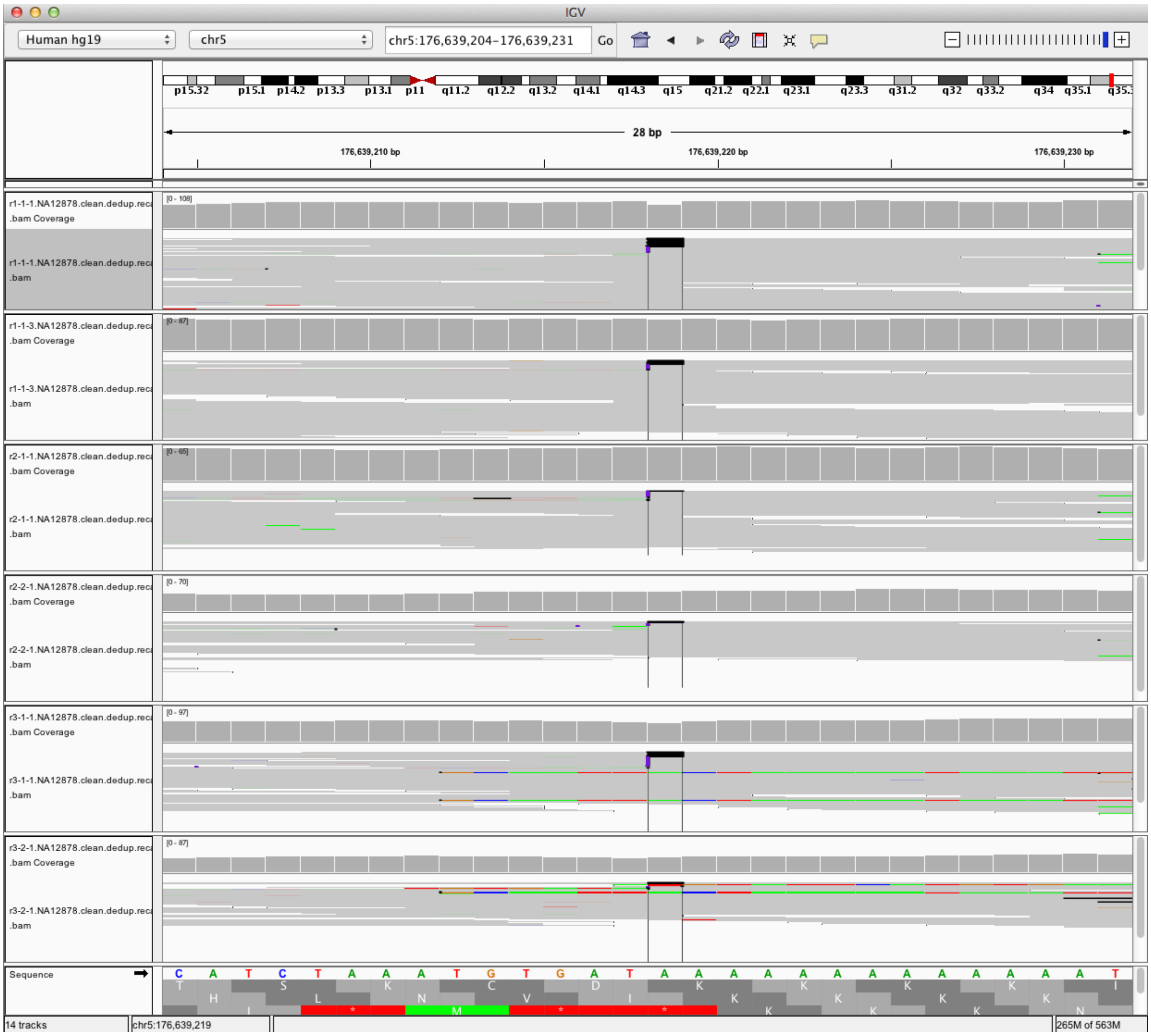

Figure 2: Example pileup for WGS replicates at chr5:176639217TA>T

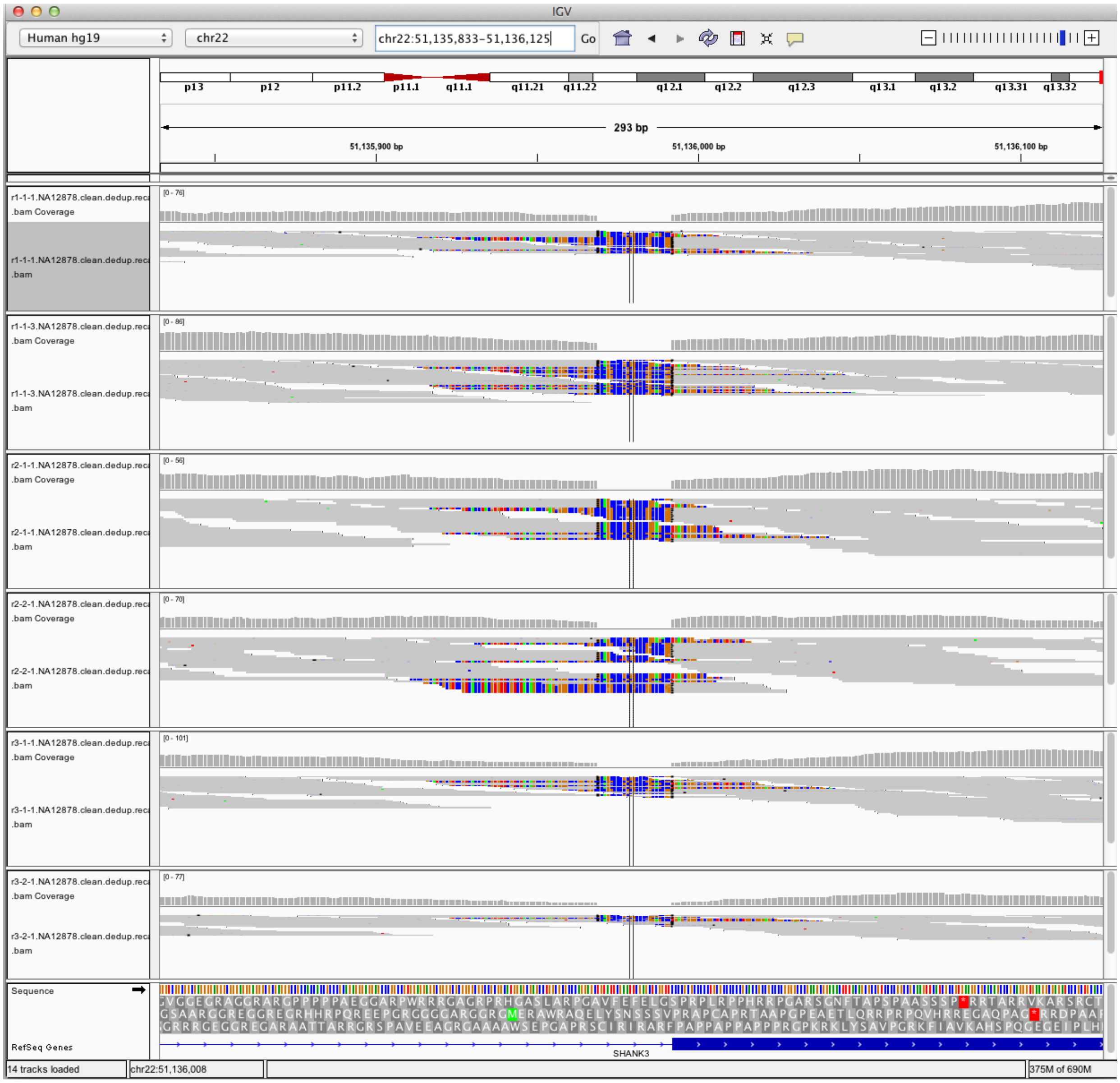

Figure 3: Structural variant in SHANK3 (chr22:51135833-51136125) that creates many false positives

Inter-Replicate Concordance Regression Analysis

Additional Tables

Table 3 and Table 4 (at the end of the supplemental) show the different comparison types for each pairs of WES and WGS technical replicates respectively.

To quantitatively assess the contribution of the different kinds of inter-replicate comparisons to the concordance, we performed a multiple linear regression analysis using the `lm` function in the R statistical package of the five different comparison kinds as binary variables and the sample ID as a binary control variable on concordance for all NA12878 and NA18507 replicate pairs. The specific model is (in R syntax):

concordance ~ Sample + intra.run + inter.run + inter.machine + inter.mode + inter.library

We assumed the behavior of concordance is linear across the narrow range of values observed in our data: WES (.970-.989) and WGS (.989-.990). Under the null hypothesis that the concordance does not vary with any comparison type, but does with the sample, the full model did not significantly differ from the null model for WES, F-statistic 0.72 (p-value = 0.61), as assessed using an F-test via the `anova` function in the R statistical package. The full model did significantly differ for WGS, F-statistic 4.69 (p-value 0.016) at a threshold of 0.05.

Based on the significant difference for WGS, we further analyzed the WGS component parameters individually. We built four models of the form:

concordance ~ Sample + <parameter>

for each of *intra.run*, *inter,run*, *inter.machine*, and *inter.library* (note that there is no *inter.machine* comparison for WGS). Using the same null hypothesis, we evaluated the significance of each individual model using an F-test. The results are summarized in Table 2. Only the *inter.library* model differed significantly at a threshold of 0.0125 (Bonferroni corrected for the 4 tests).

Table 2: Regression coefficients for WGS replicate comparisons

Coefficient	F-statistic	P value
**intra.run**	0.4286	0.5226
**inter.run**	2.25	0.1544
**inter.machine**	1.8	0.1997
**inter.library**	14.7	0.001626

References

1. DePristo MA, Banks E, Poplin R, Garimella K V, Maguire JR, Hartl C, Philippakis AA, del Angel G, Rivas MA, Hanna M, McKenna A, Fennell TJ, Kernytsky AM, Sivachenko AY, Cibulskis K, Gabriel SB, Altshuler D, Daly MJ: **A framework for variation discovery and genotyping using next-generation DNA sequencing data.** *Nature genetics* 2011, **43**:491–8.

2. McKenna A, Hanna M, Banks E, Sivachenko A, Cibulskis K, Kernytsky A, Garimella K, Altshuler D, Gabriel S, Daly M, DePristo MA: **The Genome Analysis Toolkit: a MapReduce framework for analyzing next-generation DNA sequencing data.** *Genome research* 2010, **20**:1297–303.

3. Li H, Durbin R: **Fast and accurate short read alignment with Burrows-Wheeler transform.** *Bioinformatics (Oxford, England)* 2009, **25**:1754–60.

4. Picard Team: **Picard**. 2012.

5. Cingolani P, Platts A, Wang LL, Coon M, Nguyen T, Wang L, Land SJ, Lu X, Ruden DM: **A program for annotating and predicting the effects of single nucleotide polymorphisms, SnpEff: SNPs in the genome of Drosophila melanogaster strain w1118; iso-2; iso-3.** *Fly* 2012, **6**:80–92.

6. Quinlan AR, Hall IM: **BEDTools: a flexible suite of utilities for comparing genomic features.** *Bioinformatics (Oxford, England)* 2010, **26**:841–2.

Additional Tables

Table 3: Comparison types for all pairs of WES technical replicates

Test	Truth	Sample	intra-run	inter-run	inter-machine	inter-mode	inter-library
r1-1-1	r1-1-2	NA12878	1	0	0	0	0
r1-1-1	r2-1-1	NA12878	0	1	0	0	0
r1-1-1	r3-1-1	NA12878	0	1	0	0	1
r1-1-1	r3-2-1	NA12878	0	1	1	0	1
r1-1-1	r5-1-1	NA12878	0	1	0	1	1
r1-1-1	r5-2-1	NA12878	0	1	1	1	1
r1-1-2	r1-1-1	NA12878	1	0	0	0	0
r1-1-2	r2-1-1	NA12878	0	1	0	0	0
r1-1-2	r3-1-1	NA12878	0	1	0	0	1
r1-1-2	r3-2-1	NA12878	0	1	1	0	1
r1-1-2	r5-1-1	NA12878	0	1	0	1	1
r1-1-2	r5-2-1	NA12878	0	1	1	1	1
r2-1-1	r1-1-1	NA12878	0	1	0	0	0
r2-1-1	r1-1-2	NA12878	0	1	0	0	0
r2-1-1	r3-1-1	NA12878	0	1	0	0	1
r2-1-1	r3-2-1	NA12878	0	1	1	0	1
r2-1-1	r5-1-1	NA12878	0	1	0	1	1
r2-1-1	r5-2-1	NA12878	0	1	1	1	1
r3-1-1	r1-1-1	NA12878	0	1	0	0	1
r3-1-1	r1-1-2	NA12878	0	1	0	0	1
r3-1-1	r2-1-1	NA12878	0	1	0	0	1
r3-1-1	r3-2-1	NA12878	0	0	1	0	0
r3-1-1	r5-1-1	NA12878	0	1	0	1	0
r3-1-1	r5-2-1	NA12878	0	1	1	1	0
r3-2-1	r1-1-1	NA12878	0	1	1	0	1
r3-2-1	r1-1-2	NA12878	0	1	1	0	1
r3-2-1	r2-1-1	NA12878	0	1	1	0	1
r3-2-1	r3-1-1	NA12878	0	0	1	0	0
r3-2-1	r5-1-1	NA12878	0	1	1	1	0
r3-2-1	r5-2-1	NA12878	0	1	0	1	0
r5-1-1	r1-1-1	NA12878	0	1	0	1	1
r5-1-1	r1-1-2	NA12878	0	1	0	1	1
r5-1-1	r2-1-1	NA12878	0	1	0	1	1
r5-1-1	r3-1-1	NA12878	0	1	0	1	0
r5-1-1	r3-2-1	NA12878	0	1	1	1	0
r5-1-1	r5-2-1	NA12878	0	0	1	0	0
r5-2-1	r1-1-1	NA12878	0	1	1	1	1
r5-2-1	r1-1-2	NA12878	0	1	1	1	1

r5-2-1	r2-1-1	NA12878	0	1	1	1	1
r5-2-1	r3-1-1	NA12878	0	1	1	1	0
r5-2-1	r3-2-1	NA12878	0	1	0	1	0
r5-2-1	r5-1-1	NA12878	0	0	1	0	0
r2-1-2	r2-1-3	NA18507	1	0	0	0	1
r2-1-2	r3-1-2	NA18507	0	1	0	0	0
r2-1-2	r5-1-2	NA18507	0	1	0	1	0
r2-1-2	r5-2-2	NA18507	0	1	1	1	0
r2-1-3	r2-1-2	NA18507	1	0	0	0	1
r2-1-3	r3-1-2	NA18507	0	1	0	0	1
r2-1-3	r5-1-2	NA18507	0	1	0	1	1
r2-1-3	r5-2-2	NA18507	0	1	1	1	1
r3-1-2	r2-1-2	NA18507	0	1	0	0	0
r3-1-2	r2-1-3	NA18507	0	1	0	0	1
r3-1-2	r5-1-2	NA18507	0	1	0	1	0
r3-1-2	r5-2-2	NA18507	0	1	1	1	0
r5-1-2	r2-1-2	NA18507	0	1	0	1	0
r5-1-2	r2-1-3	NA18507	0	1	0	1	1
r5-1-2	r3-1-2	NA18507	0	1	0	1	0
r5-1-2	r5-2-2	NA18507	0	0	1	0	0
r5-2-2	r2-1-2	NA18507	0	1	1	1	0
r5-2-2	r2-1-3	NA18507	0	1	1	1	1
r5-2-2	r3-1-2	NA18507	0	1	1	1	0
r5-2-2	r5-1-2	NA18507	0	0	1	0	0

Table 4: Comparison types for all pairs of WGS technical replicates

Test	Truth	Sample	intra-run	inter-run	inter-machine	inter-mode	inter-library
r1-1-1	r1-1-3	NA12878	1	0	0	0	0
r1-1-1	r2-1-1	NA12878	0	1	0	0	0
r1-1-1	r2-2-1	NA12878	0	1	1	0	0
r1-1-1	r3-1-1	NA12878	0	1	0	0	1
r1-1-1	r3-2-1	NA12878	0	1	1	0	1
r1-1-3	r1-1-1	NA12878	1	0	0	0	0
r1-1-3	r2-1-1	NA12878	0	1	0	0	0
r1-1-3	r2-2-1	NA12878	0	1	1	0	0
r1-1-3	r3-1-1	NA12878	0	1	0	0	1
r1-1-3	r3-2-1	NA12878	0	1	1	0	1
r2-1-1	r1-1-1	NA12878	0	1	0	0	0
r2-1-1	r1-1-3	NA12878	0	1	0	0	0
r2-1-1	r2-2-1	NA12878	0	0	1	0	0

r2-1-1	r3-1-1	NA12878	0	1	0	0	1
r2-1-1	r3-2-1	NA12878	0	1	1	0	1
r2-2-1	r1-1-1	NA12878	0	1	1	0	0
r2-2-1	r1-1-3	NA12878	0	1	1	0	0
r2-2-1	r2-1-1	NA12878	0	0	1	0	0
r2-2-1	r3-1-1	NA12878	0	1	1	0	1
r2-2-1	r3-2-1	NA12878	0	1	0	0	1
r3-1-1	r1-1-1	NA12878	0	1	0	0	1
r3-1-1	r1-1-3	NA12878	0	1	0	0	1
r3-1-1	r2-1-1	NA12878	0	1	0	0	1
r3-1-1	r2-2-1	NA12878	0	1	1	0	1
r3-1-1	r3-2-1	NA12878	0	0	1	0	0
r3-2-1	r1-1-1	NA12878	0	1	1	0	1
r3-2-1	r1-1-3	NA12878	0	1	1	0	1
r3-2-1	r2-1-1	NA12878	0	1	1	0	1
r3-2-1	r2-2-1	NA12878	0	1	0	0	1
r3-2-1	r3-1-1	NA12878	0	0	1	0	0
r2-1-3	r2-1-4	NA18507	1	0	0	0	1
r2-1-3	r3-1-3	NA18507	0	1	0	0	0
r2-1-4	r2-1-3	NA18507	1	0	0	0	1
r2-1-4	r3-1-3	NA18507	0	1	0	0	1
r3-1-3	r2-1-3	NA18507	0	1	0	0	0
r3-1-3	r2-1-4	NA18507	0	1	0	0	1
